# Long Non-Coding RNAs in Atrial Fibrillation: Pluripotent Stem Cell-Derived Cardiomyocytes as a Model System

**DOI:** 10.3390/ijms21155424

**Published:** 2020-07-30

**Authors:** Emre Bektik, Douglas B. Cowan, Da-Zhi Wang

**Affiliations:** 1 Department of Cardiology, Boston Children’s Hospital, Harvard Medical School, 300 Longwood, Boston, MA 02115, USA; Emre.Bektik@childrens.harvard.edu (E.B.); Douglas.Cowan@childrens.harvard.edu (D.B.C.); 2 Harvard Stem Cell Institute, Harvard University, Cambridge, MA 02138, USA

**Keywords:** atrial fibrillation, heart disease, arrhythmia, long non-coding RNAs, pluripotent stem cells, iPSC disease modeling

## Abstract

Atrial fibrillation (AF) is a type of sustained arrhythmia in humans often characterized by devastating alterations to the cardiac conduction system as well as the structure of the atria. AF can lead to decreased cardiac function, heart failure, and other complications. Long non-coding RNAs (lncRNAs) have been shown to play important roles in the cardiovascular system, including AF; however, a large group of lncRNAs is not conserved between mouse and human. Furthermore, AF has complex networks showing variations in mechanisms in different species, making it challenging to utilize conventional animal models to investigate the functional roles and potential therapeutic benefits of lncRNAs for AF. Fortunately, pluripotent stem cell (PSC)-derived cardiomyocytes (CMs) offer a reliable platform to study lncRNA functions in AF because of certain electrophysiological and molecular similarities with native human CMs. In this review, we first summarize the broad aspects of lncRNAs in various heart disease settings, then focus on their potential roles in AF development and pathophysiology. We also discuss current uses of PSCs in AF research and describe how these studies could be developed into novel therapeutics for AF and other cardiovascular diseases.

## 1. Introduction

Atrial fibrillation (AF) is the most common form of cardiac arrhythmias among heart patients [1], which ultimately leads to heart failure and other complications, which increase morbidity and mortality [2]. The pathology of AF is characterized by remodeling of the atrial electrical system—often mediated by abnormalities in ion channels [3,4,5]. One major feature of atrial remodeling in AF is the shortened refractory period leading to reentry due to reduced action potential duration (APD) [6,7]. The cause of AF is not fully understood, as genetic background, lifestyle, hypertension, or other underlying pathological conditions can all play a role [8].

With the advancement of new RNA sequencing technologies, a large set of noncoding RNAs (ncRNAs) were characterized in AF patients, which were believed to have essential roles in both atrial development and disease [9]. NcRNAs are non-protein coding RNAs that are divided into two major groups: small ncRNAs less than 200 nt in length and long ncRNAs (lncRNAs) more than 200 nt in length. MicroRNAs (miRNAs), a subgroup of short non-coding RNAs, have been widely studied in AF and shown to play an essential role in this condition [10,11]. In past decade, the identification of lncRNAs opened a new avenue of investigation in AF research. Accumulating evidence shows that lncRNAs play key roles in several cardiovascular diseases, including AF [12]. Consequently, it is important to determine how they function in AF development and whether they could be used to treat AF. Indeed, recent studies have shown that lncRNAs can serve as molecular biomarkers in AF patients and possess the potential to be therapeutic targets [13,14]. Therefore, the identification and characterization of new lncRNAs could shed light on the mechanisms behind AF and may serve to prevent, diagnose, and, ultimately, treat AF. Because the causes of AF are highly variable between human and animal models, it is essential to develop appropriate disease models using human cells or tissues [15]. Human pluripotent stem cells (hPSCs) are emerging as an essential system to model AF, whether they are derived from patients or genetically manipulated in vitro.

In this review, we discuss the studies that outline the role of lncRNAs in heart diseases such as AF, present disease-modeling approaches for AF, and discuss the experimental and conceptual challenges associated with these studies with an emphasis on improving disease modeling.

## 2. Pathophysiological Remodeling of Atria

Pathophysiological atrial remodeling affects electrical conduction and the structure of these cardiac chambers as well as their contractile properties, which, eventually, results in the development of arrhythmia. As a result, atrial remodeling is a major determinant of AF.

### 2.1. Electrical Remodeling

Electrical remodeling is often associated with an alteration in the expression or function of ion channels, promoting atrial arrhythmogenesis. AF and rapid tachyarrhythmias are a major cause of electrical remodeling [16,17,18]. As Ca^2+^ enters atrial myocytes during each cycle of action potential (AP) generation, rapid pacing elevates Ca^2+^ levels that, in turn, initiates an autoprotective mechanism by decreasing Ca^2+^ by shortening action potential duration (APD) [17,19,20]. Shortened APD, as a result of changes in Ca^2+^ and K^+^ currents, stabilizes atrial reentrant cycles and increase vulnerability and sustainability of AF [17,19,20,21]. These alterations promote Ca^2+^ release during diastole, leading to ectopic electrical activity [17,20]. In addition, there are several clinical phenomena that contribute to electrical remodeling in the atria, which include early AF recurrence post-cardioversion, drug resistance in prolonged AF, and progression from paroxysmal AF attacks to persistent ones [22].

### 2.2. Structural Remodeling

Fibrosis is the main structural remodeling response associated with most forms of AF [16,17,20,23,24,25]. Muscle bundles are separated by interstitial fibrosis, while dead atrial cardiomyocytes are replaced by reparative fibrosis, which interferes with, and slows down, electrical conduction [23,24]. Fibrosis causes progression from paroxysmal to permanent AF; therefore, the fibrotic response is a potential therapeutic target for the treatment of AF [16,23,25] as well as an indicator for the need of treatment [26]. Either way, fibroblast function is likely altered by AF, leading to structural remodeling [27] and creating a positive feedback loop between AF and fibrosis, which establishes permanent AF.

### 2.3. Contractile Remodeling

Atrial contractile function is reduced by the electrical and structural alterations caused by AF. Atrial contractile remodeling has been recognized for decades [28]; yet, the mechanisms driving contractile dysfunction remain to be fully elucidated. Abnormal Ca^2+^ handling in atria greatly contributes to the contractile dysfunction, which primarily result from alterations in trans-membrane Ca^2+^ transport. In addition to alterations in Ca^2+^ handling, changes to the contractile proteins contribute to reduced contractility. Previous studies showed that the expression and phosphorylation status of contractile proteins were changed in AF patients [29]. Myosin, a component of the contractile filaments, has two subtypes: alpha-myosin heavy chain (αMHC), which has faster contraction rates, and beta-myosin heavy chain (βMHC), which has slower contraction rates. Atria predominantly expresses αMHC with much lower expression levels of βMHC [30]; however, βMHC levels are elevated in chronic AF [31,32]. The atrial light chain components of the MHC, which influence maximum force-generating capacity of the myosin head, were down-regulated in AF patients [29]. In addition, the phosphorylation of cardiac troponin T (cTnT) was increased in AF patients, which might be a reason for the observed decreases in maximal contractile force. Thus, changes in the maximal force-generating contractile apparatus as well as alterations in Ca^2+^ handling and myolysis of myosin proteins can contribute to decreased contractility in AF.

## 3. lncRNAs and Their Interventions in Heart Disease

### 3.1. Features and Mechanisms of lncRNAs

Long non-coding RNAs (lncRNAs) are classified as RNAs >200 nt in length that are encoded by genomic loci within or between protein-coding genes [33,34]. Although many lncRNAs lack protein-coding signatures (therefore referred to as non-coding), some may contain small open reading frames (sORFs) [35,36,37], which can be occupied by the translational machinery [38] and encode functional small peptides (<100 aa) [39]. lncRNAs are generated and processed similar to messenger RNAs (mRNAs), which include epigenetic chromatin marks, transcription by RNA polymerase II, 5′ capping, 3′ poly-adenylation, and splicing [40,41]. In addition, lncRNAs have promoter regions that contribute to their widespread expression throughout the genome [42].

Based on their genomic proximity to target encoding regions, lncRNAs are classified into five major groups: (1) Intronic lncRNAs, which are transcribed entirely from intronic regions of protein-coding genes; (2) intergenic lncRNA, which are independently transcribed between protein-coding genes from either sense or antisense DNA strands; (3) sense lncRNAs, which overlap exons or introns of mRNAs; (4) antisense lncRNAs, which are transcribed from the opposite strand of protein-coding genes; and (5) bidirectional lncRNAs, which share promoters with protein-coding genes, but are transcribed in the opposite direction [43,44,45].

The function of lncRNAs are not fully understood. Because lncRNAs may be comprised of thousands of nucleotide sequences, and they form complex and flexible secondary structures with higher-order motifs that can interact with DNA, RNA, and proteins [46]. Their unique structures and cellular localization in the nucleus or cytoplasm determine their function. Nuclear lncRNAs are involved in the transcriptional and epigenetic regulation of neighboring genes (cis-action) or distal genes (trans-action), influencing gene expression by helping to assemble the chromatin remodeling machinery. Some lncRNAs are involved in the repression of gene expression by sequestering RNA-binding proteins or transcription factors, while some enhance gene activation by mediating the demethylation of promoters, activation of transcription factors, or modification of the chromosomal structure. Moreover, certain lncRNAs may directly interact with, and regulate, the transcriptional machinery as well as posttranscriptional processes, such as RNA editing and splicing. On the other hand, cytoplasmic lncRNAs are involved in translation, stability, and decay of mRNAs. They can act as decoys or sponges for protein complexes in the cytosol or mediate the function of miRNAs by targeting and repressing the translation of mRNAs [40,47,48]. lncRNAs that are involved in the regulation of heart development and function are listed in Table 1.

### 3.2. Functional Roles of LncRNAs in the Heart

#### 3.2.1. LncRNAs in the Developing Heart

LncRNAs are expressed at much lower levels than protein-coding genes, yet many lncRNAs play essential and diverse roles in mammals [96,97,98]. Of relevance to this review, lncRNAs have been shown to play crucial roles in heart development. Although their functions in gene regulatory networks during development are still under investigation, some important players have been identified. One of the first-discovered and well-studied lncRNAs in the heart is Braveheart (Bvht) [49,99]. Klattenhoff et al. [49] established the role of Bvht in the lineage commitment of mouse embryonic stem cells (ESCs) to the cardiac mesoderm. They showed that deletion of Bvht in ESCs altered the expression of 548 genes, including the cardiac transcription factors Mesp-1, Gata4, Tbx5, Hand1, Hand2, and Nkx2.5. Bvht acts as upstream of Mesoderm posterior-1 (Mesp1), which is transiently expressed in the nascent mesoderm during differentiation of the cardiac lineage. They also showed that Bvht interacts with SUZ12, a component of the polycomb repressive complex-2 (PRC2), in differentiating cardiomyocytes to epigenetically regulate cardiac lineage commitment [49].

Similar to Bvht, the cardiac mesoderm enhancer-associated non-coding RNA (CARMEN) also interacts with components of the PRC2 complex (EZH2 and SUZ12) to contribute to cardiac specification and differentiation. Interestingly, silencing of CARMEN inhibits Bvht expression, suggesting that CARMEN acts upstream of Bvht. Nevertheless, CARMEN also activates core cardiac transcriptional regulators as well as genes encoding contractile proteins such as Gata4, Tbx5, Nkx2.5, Myh6, and Myh7 [50].

Another regulator of Mesp1 during heart development is the long intergenic non-coding RNA-1405 (lnc1405), which recruits Eomes, a transcription factor involved in developmental progression, to the Mesp1 promoter. Lnc1405 also physically mediates the interaction of Eomes with the Trithorax group (TrxG) subunit WDR5 and the histone acetyltransferase GCN5 at the Mesp1 promoter and contributes to cardiogenesis by activation of the cardiac differentiation network in vivo [51].

The fethal-lethal non-coding developmental regulatory RNA (Fendrr) is a lncRNA restricted to mesoderm that acts upstream of the Forkhead box 1 (Foxf1) transcription factor. Fendrr occupies the promoter region of Foxf1 and epigenetically silences it by modulating PRC2 and TrxG complexes, leading to increased H3K27me3. In contrast, Fendrr knockdown in embryonic stem cells (ESCs) increased the expression of Gata6 and Nkx2.5 by increasing H3K4me3 occupation at their promoters [52].

PITX2 adjacent non-coding RNA (PANCR) is a lncRNA that controls expression of PITX2, a critical factor in determining left-right organ patterns during development [100,101]. With respect to the heart, PANCR knockdown results in decreased expression of PITX2 in cardiomyocytes derived from ESCs, suggesting that PITX2 expression relies on PANCR [53]. Pitx2 locus-asymmetric regulated RNA (Playrr) is another lncRNA that interacts with Pitx2. Expression of Pitx2 and Playrr affects left or right organ development—Playrr is active on the right side and suppresses Pitx2 expression, while Pitx2 is expressed on the left and silences Playrr expression [54].

Hand2, a transcription factor central in heart development, has been shown to be regulated by a pair of lncRNAs, Upperhand (Uph or Hand2os1) and Handdown (Hdn), which are upstream and downstream of the Hand2 locus, respectively. Uph shares a bidirectional promoter with Hand2 and is essential for transcriptional regulation of Hand2 through recruitment of Gata4 to the superenhancer locus of Upd-Hand2. Uph knock-out mice developed right ventricular hypoplasia and died perinatally because of abolished Hand2 expression, which suggests an essential role for the Upd-Hand2 interaction in heart development [55]. On the other hand, Hdn regulates early cardiac development by suppressing Hand2 expression. Transcriptional activity from both the Upd and Hdn loci, but not their mature transcripts, oppositely regulate Hand2 expression [55,56,102].

LncRNA HA117, which is known to suppress cardiomyocyte differentiation, is associated with diminished cardiac function in children with Tetralogy of Fallot (TOF). TOF is a common congenital heart defect usually characterized with a narrowed right ventricular outflow tract (RVOT) due to abnormal cardiomyocyte differentiation [57]. Although the functional contribution of HA117 to the pathogenesis of TOF is yet to be elucidated, expression of this lncRNA may prove to be a useful prognostic indicator [57].

In addition, lncRNAs have important functions for cardiogenesis as well as maturation, contractility, electric conduction, etc. For example, Luther et al. [103] demonstrated that an antisense lncRNA in neonatal rat cardiomyocytes inhibited expression of both αMHC and βMHC. Varying the level of antisense lncRNA expression alters the levels of αMHC and βMHC, which modifies contractility in cardiac myocytes. Relatively higher levels of αMHC accentuates contractile force, whereas higher levels of βMHC is linked with slower and more efficient contraction. The findings by Luther et al. [103] suggest that the antisense lncRNA regulates switching between αMHC and βMHC isoforms by opposing the expression of both.

#### 3.2.2. LncRNA in the Failing Heart

The myocardial transcriptome, including lncRNAs, is dynamically regulated in the failing heart. High-throughput RNA sequencing technologies have permitted the identification of dysregulated lncRNAs in heart failure (HF) in animal models and in humans [60,61,104,105,106,107,108]. In the past decade, lncRNA have been shown to play critical roles in various pathological conditions in the heart (e.g., hypertrophy, myocardial infarction, fibrosis, and cardiac arrhythmias), which constitute potential therapeutic targets (Table 1). Increased global mortality due to heart disease attracted efforts toward investigating this class of non-coding RNAs with the hope of developing novel therapeutics; therefore, a detailed understanding of lncRNA function in the heart disease is necessary. Below, we review many of the known lncRNAs involved in cardiovascular disease.

##### Cardiac Hypertrophy

Pathological hypertrophy is initially induced as a compensatory response to cardiac stress or injury, and is usually accompanied by cardiomyocyte apoptosis, activation of a fetal gene expression program, and fibrosis, which ultimately lead to chamber dilation and heart failure [109]. Myosin heavy chain associated RNA transcripts (Mhrt), a group of lncRNAs derived from the Myh7 genomic loci, have been discovered to protect heart against hypertrophy [58,110]. Mhrt is downregulated in a mouse model of cardiac hypertrophy induced by thoracic aortic constriction (TAC). Conversely, overexpression of Mhrt reversed the hypertrophic response elicited by TAC. Mhrt was found to function through competitive binding to Brg1 in the Brg1-Hdac-Parp chromatin repressor complex; thereby, preventing Brg1 from initiating an isoform switch from Mhy6 to Myh7. This mechanism seems to be conserved in humans. Alternatively, Mhrt suppressed expression of myocardin, a muscle-specific transcriptional co-activator, by reducing myocardin acetylation and inhibiting hypertrophy-related genes regulated by myocardin [59]. Also, myocardin increased transcriptional activity of Mhrt by binding the Mhrt promoter, generating a positive feedback loop. These studies suggest an essential role for Mhrt in cardiac hypertrophy.

Another lncRNA identified using the TAC-induced hypertrophic mouse model is cardiac hypertrophy-associated transcript (Chast) [60]. Silencing of Chast attenuated the hypertrophic response in the TAC model, while overexpression induced hypertrophy in vitro and in vivo. The nuclear factor of activated T cells (NFAT) acts as upstream activator of Chast, which subsequently downregulates the Pleckstrin homology domain-containing protein family M member 1 (Plekhm1), a cardiac autophagy regulator, and leads to abnormal remodeling and hypertrophy. This mechanism is conserved in humans.

Cardiac-Hypertrophy-Associated epigenetic regulator (Chaer) is a lncRNA identified through transcriptome analyses on pressure-overload-induced hypertrophic mice. Chaer interacts with PRC2 via the EZH2 binding motif and interferes with targeting action of PRC2 on promoter regions from hypertrophy-related genes [61]. The Chaer-PRC2 interaction disrupts the suppressive methylation, H3K27me3, which in turn upregulates pro-hypertrophic gene expression (Anf, Myh7, Acta1, etc.). On the other hand, suppression of Chaer reduced cardiac remodeling only at the early stage of TAC-induced hypertrophy, suggesting a critical window for epigenetic regulation by the Chaer-PRC2 complex.

Apart from aforementioned lncRNAs that modulate epigenetic regulation of the hypertrophic response, some lncRNAs mediate hypertrophic processes by regulating miRNAs via altering stability or targeting of these molecules. One of the key lncRNAs identified in cardiac hypertrophy is cardiac hypertrophy-related factor (CHRF), which functions by suppressing the anti-hypertrophic miR-489/Myd88 [63] or miR-93/Akt3 axis [62]. Another extensively studied lncRNA is myocardial infarction-related transcripts (MIAT), which was found to regulate hypertrophy through sponging anti-hypertrophic miR-150 [64]. In a later study, the MIAT/miR-150 axis was shown to induce hypertrophy via the downstream effector P300 [111]. MIAT was also found to regulate the miR-93/TLR4 axis, where sponging of miR-93 by MIAT induces TLR4 expression and hypertrophic PI3K/Akt/mTOR signaling, leading to AngII-induced hypertrophy [65]. A group of other lncRNAs that regulate miRNAs in hypertrophy are H19 [66], lncRNA-ROR [67], HOTAIR [68], DSCAM-AS1 [69], Plscr4 [70], XIST [71,72], SYNE1-AS1 [73], MAGI1-IT1 [74].

##### Myocardial Infarction

Myocardial infarction generates a complicated remodeling process that includes fibrosis—ultimately causing ischemic heart failure [112]. LncRNAs may play a key role in this process. An initial study showed that the lncRNA cardiac autophagy inhibitor factor (CAIF) has a cardio-protective effect by suppressing autophagy and attenuating the effects of myocardial infarction via interactions with the p53/myocardin axis to block transcriptional activation of myocardin [75]. In an ischemic heart failure study, β-secretase-1 (BACE1), an enzyme producing β-amyloid peptide, was shown to have an antisense lncRNA transcript BACE1-AS, which stabilizes the BACE1 transcript [76]. BACE1-AS upregulates BACE1 expression, leading to the accumulation of β-amyloid protein, which is toxic for cardiomyocytes and endothelial cells. These findings suggest that BACE1-AS contributes to the pathogenesis of ischemic heart failure.

There is also a group of lncRNAs that exert their function in myocardial infarction by suppressing miRNA function. For example, the cardiac apoptosis-related lncRNA (CARL) was found to suppress mitochondrial fission and apoptosis in cardiomyocytes through sponging of miR-539 and, thereby, inhibiting PHB2, an inhibitor of mitochondrial fission and apoptosis [77]. Therefore, the inhibition of CARL may point toward a therapeutic approach to increase the survival of cardiomyocytes after myocardial infarction and preserve heart function. Another miRNA-targeting lncRNA is autophagy promoting factor (APF) that induces adaptive cell autophagy in cardiomyocytes by targeting the miR-188-3p/ATG7 axis [78]. APF was found to bind to and suppress the inhibitory effect of miR-188-3p on ATG7, which results in increased ATG7 levels and enhanced cardiomyocyte autophagy within the infarcted region. Inhibition of APF in animal models significantly attenuated autophagy and decreased infarct size in response to ischemia/reperfusion (I/R) injury.

Aside from apoptosis and autophagy, some miRNA-targeting lncRNAs can function in necrosis. For instance, Necrosis-related factor (NRF) is involved in cardiomyocyte necrosis by acting as an endogenous sponge RNA for miR-873, a miRNA which inhibits necrotic cell death induced by H_2_O_2_ or I/R injury in mouse hearts via inhibition of receptor-interacting serine/threonine-protein kinase 1 and 3 (RIPK1/RIPK3) [79]. Activation of NRF, by its upstream target p53, inhibits miR-873 expression by direct binding, resulting in increased expression of RIPK1/RIPK3 and induction of cardiomyocyte necrosis. In addition, there are other lncRNAs identified in infarcted hearts such as Meg3, which induce apoptosis by interacting with the RNA-binding protein FUS [80]. NEAT1 regulates cardiomyocyte proliferation by suppressing miR-378-3p [81], LINC01614 promotes hypoxia/re-oxygenation-induced injury by sponging miR-138-5p [82], and XIST promotes ischemic damage by targeting the miR-101a-3p/FOS axis [83].

##### Cardiac Fibrosis

Cardiac fibrosis results from fibroblast activation and differentiation into myofibroblasts, which increases extracellular matrix (ECM) deposition to replace dead myocardium [113]. This is a beneficial process in the early stages of fibrosis; yet, excessive and/or progressive accumulation of ECM components can increase tissue stiffness causing impaired cardiac function [114,115].

A cardiac fibroblast-specific lncRNA, Wisp2 super-enhancer-associated RNA (WISPER), was identified by RNA sequencing following myocardial infarction [84]. WISPER exerts its effect in fibrosis through association with TIA1-related protein and pro-fibrotic lysyl hydrolase 2. In the absence of WISPER in vivo, fibrosis was suppressed through the attenuation of proliferation and differentiation of cardiac fibroblasts into myofibroblasts, suggesting WISPER is a potential therapeutic target for suppressing cardiac fibrosis.

A fibroblast-enriched lncRNA, maternally expressed gene 3 (Meg3), was found to be downregulated in pressure overload-induced cardiac fibrosis [85]. Meg3 functions through its interactions with the p53/matrix metalloproteinase-2(MMP-2) axis. Inhibition of Meg3 transcripts in cardiac fibroblasts inhibits expression of MMP2, a mediator of cardiac fibrosis. Meg3 inhibition in vivo attenuated cardiac fibrosis and improved diastolic heart function in a pressure overload-induced model.

Metastasis Associated Lung Adenocarcinoma Transcript 1 (MALAT1), also known as nuclear enriched abundant transcript 2 (NEAT2), is a lncRNA upregulated in infarcted hearts and in angiotensin II (AngII)-activated cardiac fibroblasts. Depletion of MALAT1 restores expression of its downstream target miR-145, which is a suppressor of transforming growth factor β1 (TGF-β1), and suppresses AngII-induced fibroblast proliferation and matrix deposition [86]. MIAT, beside its role in myocardial infarction, was shown to regulate cardiac fibrosis through its interaction with another molecular mechanism. MIAT upregulation in infarcted hearts and serum or AngII-treated cultured cardiac fibroblasts was accompanied by the downregulation of miR-24 and upregulation of Furin and TGF-β1 [87]. Knockdown of MIAT in vivo reduced cardiac fibrosis, deregulated fibrosis genes, and restored heart function, suggesting MIAT is another potential therapeutic target to treat fibrosis. There are other examples of lncRNAs involved in cardiac fibrosis including n379519 [116,117], which promotes cardiac fibrosis by sponging miR-30 [88], and Crnde, which attenuates cardiac fibrosis in a negative feedback loop with Smad3 in diabetic cardiomyopathy [89].

##### Cardiac Arrhythmias

Electrical remodeling of the heart involves dysregulation or dysfunction of cardiac ion channels in response to pathological or genetic conditions, creating a proarrhythmogenic substrate and ultimately leading to arrhythmias in ventricular or atrial compartments of the heart. In recent years, lncRNAs have been shown to be involved in electrical remodeling of the heart.

*MALAT1*, besides its function in ischemic injury, was found to be upregulated following myocardial infarction-induced ventricular arrhythmias in rats [90]. MALAT1 acts as a sponge for miR-200c and inhibits its levels in rat cardiomyocytes. Overexpression of MALAT1 was shown to reduce miR-200c levels, leading to increased expression of high-mobility group box 1 (HMGB1), which decreased expression levels of Kv4.2 and Kv4.3 ion channels, dysregulating cardiac transient outward currents (I_to_) [90,118]. LncRNA Kcna2-AS was found to contribute to ventricular arrhythmias [91]. Kcna2-AS is a negative regulator of Kcna (encoding Kv1.2 potassium channel), which helps control heart rate as well as performing several other physiological functions. In failing rat ventricles, Kcna2-AS expression was found to be upregulated while Kcna2 expression was downregulated. Knockdown of Kcna2 in rat hearts resulted in reduced delayed rectifier potassium current (I_Ks_) and prolonged action potential duration. Thus, elevated levels of Kcna2-AS downregulated Kcna2 and increased ventricular arrhythmia susceptibility.

Gap junctions, which are composed of connexin (Cx) proteins, are an important component of electrical conduction in the heart. The lncRNA cardiac conduction regulatory RNA (CCRR) was shown to bind to CIP85, which interacts and promotes degradation of Cx43, and inhibits the CIP85:Cx43 interaction to increase Cx43 levels at the intercalated disc (ICD) [92]. Overexpression of CCRR reversed intercellular conduction block and contractile dysfunction by increasing levels of Cx43 at the ICD in failing mouse hearts.

The sarco/endoplasmic reticulum Ca^2+^-ATPase (SERCA) is the major component of the Ca^2+^ efflux mechanism and is an important ion pump for maintaining Ca^2+^ homeostasis. Expression of the SERCA2a cardiac isoform in heart failure is significantly reduced, leading to aberrant Ca^2+^ homeostasis. The lncRNA Z-box binding 1-type containing 1 antisense RNA1 (ZFAS1) was found to be upregulated in heart failure and to repress SERCA2a expression and activity by direct binding [93]. Inhibition of ZFAS1 canceled out the devastating effects of ZFAS1 on Ca^2+^ homeostasis in the heart.

Although lncRNAs are annotated as non-coding, previous studies showed that some lncRNA loci contain open reading frames (ORFs), which are translated into functional micropeptides [119,120,121]. Some micropeptides regulate SERCA activity in the heart. For example, a 31 amino-acid micropeptide Sarcomlamban (Scl), encoded from putative noncoding RNA 003 in 2 L (pncr003:2 L), was identified in *Drosophila*, and found to be localized to sarcoplasmic reticulum to regulate SERCA activity. Alterations in Scl expression resulted in abnormal Ca^2+^ efflux and contraction [94]. Another example is dwarf open reading frame (DWORF), a muscle-enriched micropeptide, which is encoded from the lncRNA LOC100507537 locus [95]. DWORF competitively binds SERCA, interfering with the inhibitory interaction of phospholamban (PLN) and SERCA in cardiac muscle, sarcolipin (SLN) in the atrium, or myoregulin (MLN) in fast-twitch skeletal muscle. Relatively high expression levels of DWORF increased Ca^2+^ influx by SERCA, which shortened the time interval of each contraction–relaxation cycle. In a failing heart, elevated DWORF expression attenuated SERCA2a inhibition by PLN and restored contractile function of heart [39,95].

## 4. LncRNAs in Atrial Remodeling and Development of AF

Although lncRNAs have been studied and shown to function in various heart diseases, their function in the pathogenesis of AF is not well-understood. AF development is a complicated process, yet it is primarily caused by either structural remodeling or electrical remodeling of the atria, which disrupts the contraction of atrial cardiomyocytes [122]. Table 2 summarizes known lncRNAs that participate in atrial remodeling and AF development.

### 4.1. LncRNAs in the Development of AF

A number of reports have described differentially expressed lncRNAs in AF [12,13,136,137]. Mei et al. [137] demonstrated that 182 lncRNAs were differentially expressed in left atrial tissue samples from patients with AF compared to patients with normal sinus rhythm. Ruan et al. [136] performed microarray analyses on AF patient samples to examine the expression profile of lncRNAs and found 219 differentially expressed lncRNAs compared to AF-free patients. They postulated that the lncRNAs identified in their study were responsible for AF initiation by promoting electrical remodeling and altering the renin-angiotensin system (RAS). In another study, Xu et al. [13] showed 177 differentially expressed lncRNAs in AF patients. They also analyzed co-expression profiles of lncRNAs with mRNAs and found that the transcriptional regulators GATA1, TAF7, and EBF1 were involved in expression of AF-related lncRNAs. Indeed, previous findings have shown that GATA1 and TAF1 have known roles in AF pathology [138,139]. Ke et al. [123] also performed a differential expression analysis of lncRNAs in the left and right atrium of AF patients and identified two AF-linked lncRNAs (RP11-99E15.2 and RP3-523K23.2) that regulate heat shock factor 2 (HSF2), which is an important player in hypertension-induced HF. Furthermore, Cheng et al. [124] examined lncRNA expression profiles in the left atrial appendage and left atrial tissue surrounding the pulmonary veins, where AF is believed to be initiated, and identified 94 differentially expressed lncRNAs. Among them, AK055347 was found to be the most significantly altered. Knockdown of AK055347 inhibited expression of the mitochondrial genes Cyp450, ATP synthase, and MASS51, resulting in decreased viability of H9C2 cardiomyocytes. These findings suggested that AK055347 may be a regulator of AF by affecting mitochondrial energy production.

In summary, lncRNAs have been identified as being involved in AF by establishing a connection between their expression and that of AF-related gene networks. Therefore, we will now focus on lncRNAs associated with structural and electrical remodeling in AF.

### 4.2. LncRNAs in Atrial Structural Remodeling

Fibrosis is a hallmark of structural remodeling of the atria and is a multifactorial process. LncRNAs were identified to interact with key factors associated with the development of atrial fibrosis and AF such as TGF-β1, which is the most commonly cited factor involved in fibrosis. Zhao et al. [125] found 57 differentially expressed lncRNAs in epicardial adipose tissue from AF patients compared to patients with normal sinus rhythm. Compelling evidence showed that lncRNAs secreted from epicardial adipose tissue diffuse into the myocardium and induce atrial fibrosis [125]. Another study showed that AF patients had increased amounts of adipose tissue on their myocardium and increased levels of the fibrotic markers TGF-β1 and Smad2 in this tissue [140]. Zhao et al. [125] identified multiple lncRNAs connected with protein-coding genes that are known to play role in atrial fibrosis. One of these lncRNAs was plasmacytoma variant translocation 1 (PVT1), whose expression was found to associate with genes related to lipid metabolism, inflammation, and TGF-β1-induced epithelial-to-mesenchymal (EMT) transition such as NOS3, TTC3, PDLIM1, and SP1 [125]. In particular, PVT1 expression was elevated in atrial muscle tissue from AF patients [126]. Overexpression of PVT1 induced fibrosis by acting as a sponge for miR-128-3p; thereby, regulating the miR-128-3p/Sp1/TGF-β1/Smad axis, leading to SP1-mediated activation of the TGF-β1/Smad pathway and increased production of collagen I and II. Conversely, inhibition of PVT1 reversed fibrosis.

ALK5, a downstream target of TGF-β1, is known to regulate the proliferation of cells [141], including cardiac fibroblasts. Growth inhibitory specificity (GAS5) is a lncRNA that inhibits ALK5 in cardiomyocytes [127]. GAS5 expression was decreased in atrial appendage samples from AF patients. Knockdown of GAS5 increased cell growth, while its overexpression inhibited growth of AC16 cells in vitro. Prostate cancer associated transcript-1 (PCAT1) is another lncRNA [128] that has been identified during the fibrotic remodeling of AF. PCAT1 expression levels were higher in atrial appendage samples from AF patients. PCAT1 promoted fibroblast proliferation by targeting TGF-β1. Aside from its function in cardiac hypertrophy and fibrosis, MIAT has recently been shown to play a role in AF through repression of miR-133-3p [129]. MIAT expression was elevated in the atrial tissues from a rat model of AF, while miR-133-3p expression was decreased. Knockdown of MIAT alleviated AF and reduced the duration of fibrillation episodes as well as promoting increased atrial function and suppressing cardiomyocyte apoptosis. On the other hand, MIAT knockdown was shown to suppress AF-induced atrial fibrosis.

Macrophages are also involved in atrial fibrosis. M1 macrophages arrive to the site of injury and clear the cellular debris, while M2 macrophages help regulate the tissue-healing process. It was shown that a switch from M1 to M2 macrophage types prevents cardiac remodeling and improves heart function [130]. Noncoding repressor of NFAT (NRON) is a lncRNA that is normally recruited to the promoter of interleukin-12 (IL-12), which is known to induce the switch from M2 to M1 types, leading to atrial fibrosis [130]. NRON was found to inhibit nuclear localization of NFAT, thus inhibiting IL-12, which reversed macrophage switching and decreased atrial fibrosis. Another study that examined lncRNAs associated with immune signaling, identified co-expression networks between up-regulated mRNAs and lncRNAs in lymphocytes collected from AF and non-AF patients [142]. Those networks were related to tumor necrosis factor (TNF), toll-like receptor (TLR), and NF-κβ signaling pathways. In short, lncRNAs from lymphocytes were linked with cellular processes including collagen synthesis, oxidative stress, inflammation, and apoptosis, which are all involved in the development of atrial fibrosis.

In addition to the above, increased sympathetic neuronal activity has been recorded in patients prior to the development of post-operative AF [143]. Interestingly, RAS has been shown to interact with the autonomic nervous system and is involved in neuronal remodeling. Wang et al. [131] analyzed lncRNAs in cardiac fat pads of canines with or without AF and found that aberrantly expressed lncRNAs were related to neuronal development, differentiation, and degeneration. Among the identified lncRNAs, they showed that in vivo inhibition of two lncRNAs (TCONS_00032546 and TCONS_00026102) related to neuronal remodeling, either shortened or prolonged the atrial refractory period, resulting in the increased occurrence or prevention of AF. In addition, the expression of these lncRNAs was negatively correlated with CCND1, FGF19, FGF4, FGF3, and SLC25A4 expression as well as the genes neighboring these lncRNAs. Together, these studies suggest that lncRNAs may contribute to the development of AF through RAS-mediated neuronal remodeling.

### 4.3. LncRNAs in Atrial Electrical Remodeling

LncRNAs are also involved in electrical remodeling in AF, although this connection is less well-established than their involvement with structural remodeling. The major drivers of electrical remodeling are shortening of the atrial refractory period and action potential duration. One of the genes identified in AF is PITX2, which is also associated with heart development. Holmes et al. [132] found that mice predisposed to developing AF had relatively low levels of PITX2. PITX2 affected cardiac ion channels to alter the atrial refractory period. In addition, PANCR, an upstream lncRNA targeting PITX2, was identified [53]. However, PANCR has not been directly linked with development of AF; yet, because of the role of PITX2 in AF, PANCR can be considered an AF-related lncRNA [142].

Li et al. [133] examined lncRNAs in AF and non-AF rabbits and found that silencing of the lncRNA TCONS_00075467 resulted in a shortened atrial refractory period and action potential duration. This effect was likely due to its role as a sponge for miR-328, which silences its inhibitory function on target mRNAs. Because of the lack of this lncRNA, miR-328 expression increases, resulting in downregulation of CACNA1C, an L-type calcium ion channel. Indeed, dysregulation of CACNA1C has been previously shown to be involved in the development of AF through regulation of RAS [144,145]. Activation of RAS has been shown to increase left atrial pressure via AngII in hypertension and heart failure. Moreover, prolongation of RAS activation induces high levels of angiotensin-converting enzyme (ACE) and AngII receptors, resulting in inflammation and fibrosis or structural remodeling [146].

In mice with AngII-induced AF, Shen et al. [134] identified an overexpressed lncRNA, KCNQ1 overlapping transcript 1 (KCNQ1OT1). KCNQ1OT1 acts as a sponge to miR-384, which targets and silences CACNA1C. Overexpression of KCNQ1OT1 inhibits the silencing effect of miR-384, leading to elevated CACNA1C levels and the development of AF.

Ke et al. [123] analyzed RNAseq data in left and right atrial appendages from patients with or without AF and identified key RNAs linked with AF. They predicted lncRNAs that potentially regulate adjacent protein-coding genes and found that lncRNA NPPA-AS1, RP11-99E15.2, and RP3-523K23.2 could interact with NPPA, ITGB3, and HSF2, respectively, and may be involved in the pathogenesis of AF. Particularly, NPPA-AS1 was found to be co-expressed with six contractile genes, including NPPA, PLCE1, TACR1, GSTO1, TNNC1, and TNN1, suggesting that NPPA-AS1 contributes to AF pathogenesis through the modulation of cardiac contraction.

## 5. Deciphering LncRNA Function in Atrial Fibrillation by hPSC Disease Modeling

**Figure 1:**
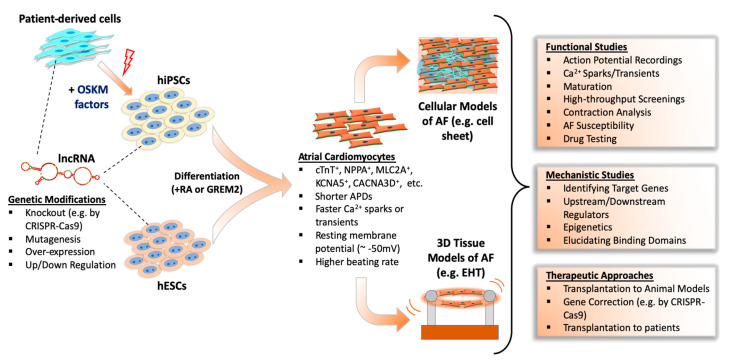
Studying function and mechanism of lncRNAs in human pluripotent stem cell-based atrial fibrillation models.

A large-scale evolutionary study showed that many lncRNAs have very limited sequential conservation unlike protein-coding counterparts [147]. Additionally, multidisciplinary analyses of lncRNAs from different species showed the vast majority of lncRNAs show minimal conservation [148]. Consequently, this limited conservation between species restricts the effective study of many lncRNAs in human AF. Also, the lack of a mechanistic understanding of AF development is an obstacle to understanding AF substrates. The most valid method is to obtain tissue samples from patients to study lncRNAs; however, there is a limited supply of these tissues from patients, let alone healthy individuals [149,150,151]. Therefore, the study of lncRNAs in AF are increasingly dependent on human pluripotent stem cell (hPSC)-derived atrial cardiomyocytes, which allows both large-scale experiments and genetic manipulation (Figure 1).

### 5.1. Differentiation and Characterization of hPSC-Derived Atrial Cardiomyocytes

Recent advances in atrial lineage differentiation of pluripotent stem cells has allowed the culture of pure and functional atrial myocytes to model AF [151,152,153,154,155,156]. Zheng et al. [153] showed that exogenous activation of retinoic acid (RA) signaling in differentiating human embryonic stem cells (hESCs) promoted an atrial phenotype as assessed by action potential characteristics and Ca^2+^ handling. Conversely, inhibition of RA signaling promoted a more ventricular differentiation program. Later, Cyganek et al. [154] further examined the molecular, cellular, and functional properties of hiPSC-derived atrial and ventricular cardiomyocytes by manipulating RA signaling in plated cells and in engineered heart muscle (EHM). By studying cell structure, action potentials, calcium fluctuations, and the transcriptome/proteome as well as contractile characteristics of EHM, they showed that the RA-dependent differentiation of hiPSC-CMs produced functionally relevant cells to model human atrial diseases. Alternatively, atrial differentiation of hESCs by a bone morphogenic protein antagonist, GREMLIN 2 (GREM 2), also caused atrial differentiation of mouse ESCs [157]; however, this method has not been validated in human ESCs despite the role of GREM2 in heart development being conserved between species [158]. Overall, these findings suggest that hPSC-derived atrial cardiomyocytes are suitable for atrial disease modeling and drug screening.

### 5.2. Disease Modeling of AF Using hPSCs

The development of atrial differentiation methods has allowed modeling of, and drug screening for, AF. For example, Laksman et al. [159] generated an AF model from hESC-derived atrial cardiomyocytes by RA activation during mesodermal differentiation. They performed optical mapping on atrial cell sheets, induced AF by rapid pacing protocol or burst pacing, and successfully showed AP propagation and reentry patterns similar to those observed in AF. Importantly, their drug testing with flecainide and dofetilide modulated reentrant arrhythmic rotor activation toward a non-AF phenotype, underscoring the reliability of these drugs in AF treatment. In a recent study by Benzoni et al. [160], a familial form of AF was modeled using patient-derived iPSC-CMs. They generated several hiPSC cell lines from patients with a persistent, untreatable AF that were not responsive to anti-arrhythmic drugs. To identify mutations in those patients, they performed whole exome sequencing and found more than 100 variations between three AF patients, of which only a few were related to genes previously linked with AF (ZFHX3) or the heart (PDE4DIP, CNN2, RYR3, NEFM, FLNC, and MYLK). Because of the complexity of AF in these patients, they decided to investigate the molecular mechanisms of AF in hiPSC-derived atrial cardiomyocytes. Functional characterization by electrophysiology analyses revealed higher beating rates due to the increased contribution of hyperpolarization activated pacemaker current (I_f_) and L-type Ca^2+^ channel current (I_CaL_) in patient-derived cardiomyocytes compared to healthy, control cells. Also, patient iPSC-CMs showed a significantly prolonged APD, and under cardiac stress, larger amplitudes of delayed after-depolarization (DAD) with more frequent ectopic beats than in control iPSC-CMs.

Hong et al. [161] also developed an AF model using patient-derived iPSCs to characterize the electrophysiological properties of a known familial AF linked with an E428K mutation on SCN5A (sodium voltage-gated alpha subunit 5). Atrial iPSC-CMs with this mutation showed an increased window for the late sodium current (I_Na,L_), increased beating rate, prolonged APD, and spontaneous arrhythmogenic activity. Interestingly, ranolazine treatment reversed the abnormal phenotypes in mutant atrial iPSC-CMs.

The above examples prove that hPSC-derived cardiomyocytes might be a helpful resource to study the function of coding or non-coding genes in AF. Although differential gene expression analyses in human atrial tissue from AF patients identified potential lncRNAs involved in AF, those studies did not fully elucidate the role of lncRNAs in the development of this disease and did not determine the molecular mechanisms underlying AF. Additionally, the limited conservation of lncRNAs between species and the variability of atrial electrophysiology and cardiac anatomy have not allowed comprehensive studies of many lncRNAs in AF, thus, limiting the translational potential of animal models. Therefore, hPSCs may offer a reliable resource to identify early mechanisms and substrates of AF, in particular, those controlled by lncRNAs.

### 5.3. Use of hPSCs for the Study of LncRNAs in AF

To date, there is a lack of reports describing the use of hPSC-CMs to study the role of lncRNAs in the development of AF; however, there are some studies that employ iPSCs to identify the molecular mechanisms of lncRNAs, which are either directly or indirectly related to AF pathogenesis.

Heart Brake LncRNA 1 (lncRNA-HBL1) was initially described in human iPSCs by Liu et al. [135] in cardiomyocyte development. LncRNA-HBL1 overexpression suppressed cardiac differentiation of iPSCs by sequestering hsa-miR-1, an essential cardiac enriched miRNA. Although this study was not directly linked to AF, previous findings showed the importance of miR-1 in AF development. miR-1 levels were reduced in the left atrium of AF patients, resulting in increased inward rectifier potassium channel (Kir2.1) [162], and miR-1 was found to accelerate shortening of the atrial effective refractory period (AERP) in a rabbit model, resulting in increased AF susceptibility by down-regulation of KCNE1 and KCNB2 [163]. These findings suggest that lncRNA-HBL1 might have a role in AF development via regulation of miR1.

NPPA-AS1 has been shown to play a role in AF via modulation of atrial contractile genes [123]. Celik et al. [164] studied the molecular mechanism of this lncRNA in iPSC-derived cardiomyocytes (iPSC-CMs), which contained a mixture of atrial, ventricular, and nodal CMs. They found that NPPA-AS1 was localized to the nucleus in iPSC-CMs. They also showed that NPPA-AS1, which is highly enriched in atrial tissue, negatively regulated NPPA expression by enhancing binding of the RE1-silencing transcription factor (REST) to the NPPA promoter, resulting in suppression of NPPA gene expression. NPPA-AS1 expression levels were relatively lower compared to NPPA levels in human atrial heart tissue, suggesting that its mode of action is limited to promoter activation, but not dimer formation with NPPA mRNAs. To evaluate the therapeutic potential of NPPA-AS1 inhibition, they tested antisense oligonucleotides (GapmeRs) in mouse hearts and showed that NPPA-AS1 silencing increased expression levels of Nppa. These observations suggest that NPPA-AS1 might be a therapeutic target to regulate NPPA expression in various heart disease conditions, including AF.

## 6. Challenges of PSC Modeling and Translational Aspects

Translation of lncRNA research requires the use of disease models in human cells or tissues as many lncRNAs are not conserved between species [165,166,167,168]. On the other hand, AF is a complex disease that shows large variations between species. Therefore, animal models have limitations for AF research [17]. Human iPSCs offer an alternative modeling platform that can help overcome some of these limitations to decipher the roles of lncRNAs in AF. At the same time, these cells have their own experimental limitations. For example, the developmental maturity of in vitro differentiated hiPSC-CMs remains a major hurdle, as they do not accurately represent the electrophysiological properties of native atrial cardiomyocytes [169]. hiPSC-CMs have been observed to have relatively depolarized resting membrane potentials during phase 4 spontaneous depolarization, decreased maximum upstroke velocity, and decreased conduction velocity [170]. Another problem is the large variation in electrophysiological (EP) properties of in vitro differentiated hESC/iPSC-CMs. Regardless of the length of culture, the EP properties show heterogeneity between different cultures, which is compounded by the utilization of various differentiation protocols [171]. Although EP properties tend to become more homogeneous with extended culture (>30 days), analysis of beating rate and EP parameters show that hESC/hiPSC-CMs are heterogenous and resemble the embryonic heart [172].

Given the facts above, results obtained from hESC/hiPSC-CMs may need to be validated in various other platforms at the moment, such as use of native human cardiomyocytes or isolated tissue samples. Indeed, recent advancements of atrial cardiomyocyte differentiation methods by manipulation of RA signaling reduced heterogeneity of atrial hESC/hiPSC-CMs in vitro, yielding atrial cardiomyocytes with EP properties resembling native atrial CMs [153,154]. Regardless, the ability to culture sufficient numbers of atrial cardiomyocytes which are effectively two-dimensional plates with the appropriate electrophysiological and contractile properties continues to present challenges for AF research and needs to be improved [151].

Recent advancements in the culture of human heart slices represents an alternative strategy to advance AF research as these tissues have a relatively long life span in optimized culture conditions; however, they come with some disadvantages such as the limited supply of healthy or diseased donors [173,174,175]. Therefore, PSC-based methods still hold promise, and improved versions such as engineered heart tissues (EHTs), may better serve the need for AF research in vitro.

EHTs are practical three-dimensional in vitro models of human heart tissue that can result in structurally and physiologically more mature cardiomyocytes. Following atrial or ventricular differentiation of hESCs/hiPSCs, cells are embedded into collagen-based hydrogels and cast into a ring or string shape to generate EHTs (Figure 1). Initially, EHTs were intended for use in heart regeneration therapies to replace dead myocardium [176]; however, they also serve as useful models to study both atrial and ventricular diseases in vitro. For example, in a recent study by Goldfracht et al. [177], ring-shaped EHTs were generated from human ESC-derived atrial cardiomyocytes. They showed these EHTs had atrial phenotypes as revealed by various validation methods, including immunostaining of atrial markers, gene expression, optical mapping of APs and CV, pharmacological testing, and mechanical force assessments. More importantly, they established an atrial-specific EHT-based arrhythmia model and validated its utility by pharmacological testing, suggesting atrial EHT models can be used for AF modeling and therapeutic drug testing.

Studying mechanistic details of lncRNAs in PSC-based cell and EHT models is an important resource for understanding AF pathophysiology. For the translation of in vitro identified AF treatment methods, a full understanding of the molecular mechanisms and safety of therapeutics is essential. However, pre-clinical animal models and non-cardiac cell models of AF resulted with limited success in clinical translation of discovered drugs as such systems are usually unable to replicate human atrial physiology. Also, the use of isolated native cardiomyocytes offer limited benefits because of their short life-span in cell culture and limited availability. The use of traditional cell culture or EHT-based tissue models of hESC/hiPSC-derived atrial cardiomyocytes may provide novel insights of AF and other diseases (Figure 1). For example, AF-related signaling pathways and their potential drug targets could be studied in hPSC-based models either through genetic modifications of PSC lines in vitro (e.g., by CRISPR-based genome editing technologies) or generating iPSC lines from patients with AF. In some cases, AF patients have inherited de novo mutations of important genes, such as ion channels or structural genes, and the effects of these mutations at the molecular and cellular levels could be extensively researched in hiPSC disease models. In addition, gene-editing technologies could be tested to reverse AF prior to animal testing and use in patients. Nonetheless, in vitro generated atrial hPSC-CMs may also serve as a resource for cell-replacement therapies in atrial diseases, including AF.

## 7. Conclusions

LncRNAs are potential candidates in the development of AF; however, the majority of lncRNAs lack sequence conservation between species and often have limited functional similarities with human AF forms because of the variations in the electrophysiological mechanisms in different species. Native human cardiomyocytes or heart tissues are the most relevant sources for studying AF in human, yet an insufficient supply and relatively short life span of these resources present a significant obstacle for AF research. The use of PSCs allows scientists to obtain human cells relatively easily and new differentiation methods permit the culture of large numbers of distinct types of human cardiomyocytes. Therefore, PSC-derived atrial cardiomyocytes should help advance research efforts to unravel the role of lncRNAs in AF pathophysiology and provide a platform to evaluate potential therapeutics.

## Figures and Tables

**Table 1:** Summary of lncRNAs involved in heart development and disease.

Heart Development		
lncRNA	Mechanism of Action	Study Model
Bvht [49]	Regulating cardiac mesoderm differentiation by targeting Mesp1 and SUZ12	Mouse ESCs
CARMEN [50]	Contributing to cardiac specification by interacting with EZH2 and SUZ12	Human CPCs; Mouse ESCs
lnc1405 [51]	Contributing to cardiogenesis by regulating Mesp1 transcription	Mouse ESCs and heart
Fendrr [52]	Regulating mesoderm specification by epigenetically silencing Foxf1	Mouse ESCs
PANCR [53]	Positively regulating expression of PITX2, contributing left organ development	Human ESC-CMs
Playrr [54]	Negatively regulating expression of Pitx2, contributing right organ development	Mouse heart and ESCs; Chick heart
Uph [55]	Acting as upstream of Hand2 and positively regulates its expression	Mouse heart
Hdn [56]	Acting as downstream of Hand2 and negatively regulates its expression	Mouse heart
HA117 [57]	Suppressor of cardiac differentiation, linked with genetic disorder TOF	Patient
**Cardiac Hypertrophy**		
Mhrt [58,59]	Protecting against hypertrophy by suppressing Brg1 and downregulating myocardin	Mouse heart (TAC); Rat CM
Chast [60]	Promoting hypertrophy by downregulating Plekhm1	Mouse heart (TAC); hESC-CM (PE)
Chaer [61]	Promoting hypertrophy via suppressing inhibitory function of PRC2 on hypertrophy genes	Mouse heart (TAC)
CHRF [62,63]	Inducing hypertrophy by suppressing the anti-hypertrophic miR-489/Myd88 or miR-93/Akt3 axis	Mouse CM (AngII or Iso); Heart (AngII or TAC)
MIAT [64,65]	Inducing hypertrophy by suppressing the anti-hypertrophic miR-150/P300 or miR-93/TLR4 axis	Mouse heart or Rat H9c2 or Rat CM (AngII)
H19 [66]	Suppressing hypertrophy by targeting the miR-675/CaMKIIδ axis	Mouse heart (TAC); Mouse CM (PE)
lncRNA-ROR [67]	Promoting hypertrophy by targeting miR-133	Mouse heart (TAC); Mouse CM (PE)
HOTAIR [68]	Suppressing hypertrophy by targeting miR-19	Mouse heart (TAC); Mouse CM (AngII)
DSCAM-AS1 [69]	Boosting hypertrophy by targeting the miR-188-5p/GRK2 axis	Mouse CMs or Rat H9c2 (AngII)
Plscr4 [70]	Attenuating hypertrophy in vitro and in vivo by targeting the miR-214/Mfn2 axis	Mouse heart (TAC) or CM (AngII)
XIST [71,72]	Preventing hypertrophy in vitro and in vivo by targeting the miR-330-3p/S100B or miR-101/TLR2 axis	Mouse heart (TAC) or CM (PE); Rat H9c2 (PE)
SYNE1-AS1 [73]	Promoting hypertrophy in vitro and in vivo by targeting the miR-525-5p/SP1 axis	Mouse heart (TAC) or CM (AngII)
MAGI1-IT1 [74]	Suppressing hypertrophy by modulating the miR-302e/DKK1/Wnt/beta-catenin axis	Rat H9c2 (AngII)
**Myocardial Infarction**		
CAIF [75]	Suppressing autophagy in infarcted CMs by blocking p53-mediated myocardin transcription	Mouse heart (I/R injury); CM (H_2_O_2_ injury)
BACE-AS1 [76]	Upregulating BACE1 transcripts that cause accumulation of β-amyloid and pathogenesis	Patient (ischemic HF); Mouse (MI)
CARL [77]	Suppressing mitochondrial fission and apoptosis through targeting the miR-359/PHB2 axis	Mouse heart (I/R injury) or CM (A/R)
APF [78]	Inducing adaptive cell autophagy through targeting the miR-188-3p/ATG7 axis	Mouse heart (I/R injury) or CM (A/R)
NRF [79]	Inducing cardiac necrosis through targeting the miR-873/RIPK1/RIPK3 axis	Mouse heart (I/R) or CM (H_2_O_2_)
Meg3 [80]	Inducing cardiomyocyte apoptosis by direct binding with RNA-binding protein FUS	Mouse heart (MI) or CM (hypoxia); hESC-CM (hypoxia) or HF Patient
NEAT1 [81]	Regulating CM proliferation through suppression of miR-378-3p	Rat Heart (I/R injury) or CM (hypoxia or H_2_O_2_); Patients (MI)
LINC01614 [82]	Promoting MI by suppression of miR-138-5p	Patient (MI); Rat H9c2 (H/R)
XIST [83]	Promoting apoptosis and MI by targeting miR-101-3p/FOS axis	Mouse Heart (MI) or CM (hypoxia)
**Cardiac Fibrosis**		
WISPER [84]	Promoting fibroblast proliferation and differentiation through activation of TIA1-related protein	Mouse heart (MI) or CF; Human CF
Meg3 [85]	Promoting cardiac fibrosis through activation of the p53/MMP2 axis	Mouse heart (TAC) or CF (TGF-β1)
MALAT1 (NEAT2) [86]	Promoting fibroblast proliferation through targeting miR-145/ TGF-β1 axis	Mouse heart (MI) or CF (Ang-II)
MIAT [87]	Promoting cardiac fibrosis through targeting miR-24/Furin/ TGF-β1 axis	Mouse heart (MI) or CF (Ang-II)
n379519 [88]	Promoting cardiac fibrosis through targeting miR-30	Rat heart (MI) or CF (TGF-β1)
Crnde [89]	Attenuating fibrosis via Smad3-Crnde negative feedback	Mouse heart (DCM) or CF (TGF-β1)
**Cardiac Arrhythmias**		
MALAT1 [90]	Overexpression dysregulates I_to_ through targeting the miR-200c/HMGB1 axis	Rat heart (MI) or CM
Kcna-AS [91]	Contributing to ventricular arrhythmias by downregulating Kcna expression	Rat heart (TAC) or CM (PE); Patient (HF)
CCRR [92]	Increasing Cx43 levels to improve intercellular cardiac conduction	Mouse heart (TAC) or CM; Patient (HF) or AC16
ZFAS1 [93]	Repressing SERCA2a expression to dysregulate Ca^2+^ homeostasis	Mouse heart (MI) or CM (hypoxia); Patient (MI) or AC16 (hypoxia)
pncr003:2 L [94]	Encoding micropeptide Sarcolamban, which regulates SERCA function	Drosophila heart
LOC100507537 [95]	Encoding micropeptide DWORF, which positively regulates SERCA activity	Mouse heart (MI) or CM; Patient (MI)

**Table 2:** LncRNAs in the development and pathophysiological remodeling of AF.

Development of AF			
LncRNA	Expression in AF	Mechanism of Action	Study Model
RP11-99E15.2 [123]	ND	May be involved in AF by regulating extracellular matrix binding via interactions with ITGB3	Patient (AF)
RP3-523K23.2 [123]	ND	May be involved in AF by regulating transcription of HSF2	Patient (AF)
AK055347 [124]	Up	Dysregulating mitochondrial energy production by regulating mitochondrial Cyp450, ATP synthase, and MSS51	Patient (AF)
**Structural Remodeling**			
PVT1 [125,126]	Up	Regulating miR-128-3p/Sp1/TGF-β1/Smad axis by sponging miR-128-3p	Patient (AF) or Atrial fibroblast; Mouse heart (Ang-II)
GAS5 [127]	Down	Inhibiting ALK5 and suppresses cell proliferation	Patient (AF) or AC16
PCAT1 [128]	Up	Promoting fibroblast proliferation through targeting TGF-β1	Patient (AF) or AC16
MIAT [129]	Up	Alleviating AF and reducing atrial fibrosis by suppressing miR-133-3p	Patient (AF); Rat (electrical stimulation)
NRON [130]	Up	Inhibiting NFAT localization to nucleus, thus suppresses IL-12 and macrophage switch from M2 to M1.	Mouse atrial CM (AngII)
TCONS_00032546 [131]	Down	Related to RAS-mediated neuronal remodeling in cardiac fat pads	Canine heart (atrial tachypacing)
TCONS_00026102 [131]	Down	Related to RAS-mediated neuronal remodeling in cardiac fat pads	Canine heart (atrial tachypacing)
**Electrical Remodeling**			
PANCR [53,132]	ND	Regulating PITX2, an AF-related gene, but not studied in AF directly	Human ESC-CM
TCONS_00075467 [133]	Down	Upregulating of it results with increased sponging of miR328, thus increasing CACNA1C levels	Rabbit right atria (AF)
KCNQ1OT1 [134]	Up	Downregulating of it results with decreased sponging of miR384, thus decreasing CACNA1C levels	Mouse heart (AngII) or CM
NPPA-AS1 [123]	Up	Modulating cardiac contraction genes (e.g., NPPA, PLCE1, TNNC1, TNN1).	Patient (AF)
lncRNA-HBL1 [135]	Up	Downregulating miR-1, an AF-related gene, but not studied in AF directly	Human iPSC-CM
